# Microfluidic Perfusion for Regulating Diffusible Signaling in Stem Cells

**DOI:** 10.1371/journal.pone.0022892

**Published:** 2011-08-04

**Authors:** Katarina Blagovic, Lily Y. Kim, Joel Voldman

**Affiliations:** 1 Research Laboratory of Electronics, Massachusetts Institute of Technology, Cambridge, Massachusetts, United States of America; 2 Health Sciences and Technology, Massachusetts Institute of Technology, Cambridge, Massachusetts, United States of America; 3 Department of Electrical Engineering and Computer Science, Massachusetts Institute of Technology, Cambridge, Massachusetts, United States of America; University of California, Merced, United States of America

## Abstract

**Background:**

Autocrine & paracrine signaling are widespread both *in vivo* and *in vitro*, and are particularly important in embryonic stem cell (ESC) pluripotency and lineage commitment. Although autocrine signaling via fibroblast growth factor-4 (FGF4) is known to be required in mouse ESC (mESC) neuroectodermal specification, the question of whether FGF4 autocrine signaling is sufficient, or whether other soluble ligands are also involved in fate specification, is unknown. The spatially confined and closed-loop nature of diffusible signaling makes its experimental control challenging; current experimental approaches typically require prior knowledge of the factor/receptor in order to modulate the loop. A new approach explored in this work is to leverage transport phenomena at cellular resolution to downregulate overall diffusible signaling through the physical removal of cell-secreted ligands.

**Methodology/Principal Findings:**

We develop a multiplex microfluidic platform to continuously remove cell-secreted (autocrine\paracrine) factors to downregulate diffusible signaling. By comparing cell growth and differentiation in side-by-side chambers with or without added cell-secreted factors, we isolate the effects of diffusible signaling from artifacts such as shear, nutrient depletion, and microsystem effects, and find that cell-secreted growth factor(s) are required during neuroectodermal specification. Then we induce FGF4 signaling in *minimal* chemically defined medium (N2B27) and inhibit FGF signaling in *fully* supplemented differentiation medium with cell-secreted factors to determine that the non-FGF cell-secreted factors are required to promote growth of differentiating mESCs.

**Conclusions/Significance:**

Our results demonstrate for the first time that flow can downregulate autocrine\paracrine signaling and examine sufficiency of extracellular factors. We show that autocrine\paracrine signaling drives neuroectodermal commitment of mESCs through both FGF4-dependent and -independent pathways. Overall, by uncovering autocrine\paracrine processes previously hidden in conventional culture systems, our results establish microfluidic perfusion as a technique to study and manipulate diffusible signaling in cell systems.

## Introduction

Autocrine and paracrine signaling are widespread both *in vivo* and *in vitro*, regulating events as diverse as tumor formation and outgrowth [1], mammalian embryogenesis [2], [3], and embryonic stem cell (ESC) pluripotency and lineage commitment [4], [5]. The spatially confined and closed-loop nature of autocrine and paracrine signaling—more generally referred to here as diffusible or soluble signaling—makes its experimental control challenging [6]. Although current experimental approaches allow known ligand-receptor interactions to be induced (*e.g.*, by adding soluble ligand or overexpressing receptor) or blocked (*e.g.*, blocking antibodies [7], small molecule inhibitors [8], receptor/ligand knockdowns or knockouts [9], [10], [11]), these specific approaches are not capable of altering diffusible signaling when the ligand/receptor pair is unknown. Altering cell density and assaying for density-dependent phenotypes is a non-specific approach commonly used to identify new loops [5], [12]. However, autocrine\paracrine loops that are sufficiently active in isolated cells may not display density-dependent phenotypes as the cell density is varied because density can only be increased above the single-cell level, further saturating the loop [6]. In order to study such loops nonspecifically, one requires a method that can effectively decrease ligand concentration to below the single-cell level.

Recently, microtechnologies have emerged that offer more precise control over cell-cell interactions. For instance, micropatterning of cells can control local cell density and hence alter diffusible signaling [13], as can use of microchannels where flow is suppressed [14], thus increasing diffusible signaling. These approaches can productively modulate diffusible signaling, but they cannot downregulate such signaling to below the single-cell level, and with both of these approaches diffusible signaling will increase over time as cells proliferate and signaling molecules accumulate. Conversely, microfluidic flow can be used to tune the relative importance of convection, diffusion and reaction [15], and is thus ideally suited to address questions where one wishes to study diffusible signaling by *removing* rather than *augmenting* ligand, such as to study tight loops.

Here we apply microfluidic perfusion to identify the existence of diffusible signaling loops in ESC processes previously hidden in conventional assays. Several reviews [16], [17], [18], [19] and prior work have suggested the use of microfluidic flow to alter and minimize autocrine\paracrine signaling [20] or to probe dose-dependent responses [21] from known exogenous factors while using perfusion to wash away cell-secreted factors, and transport models suggest that it is possible to use convection to alter extracellular ligand concentrations [22], [23]. However, to date there has been no clear demonstration of perturbing autocrine\paracrine signaling via flow in a biologically significant manner, nor has flow been used to elucidate diffusible signaling in stem cell biology.

ESCs are one biological system that illustrates both the importance of and challenges present in studying diffusible signaling. ESCs are being widely investigated both for their potential therapeutic applications (*e.g.*, regenerative medicine) and as *in vitro* models of development. Their utility depends in large part on our ability to control their fate decisions *in vitro*. One important fate choice is that of neural specification. mESCs can be readily differentiated into neuronal cells using external factors including retinoic acid [24] or Sonic hedgehog agonists [25]. mESCs can also differentiate into neural precursors in adherent monoculture in serum-free defined medium in the absence of the self-renewal factors leukemia inhibitory factor (LIF) and bone morphogenetic protein 4 (BMP4) [5]. In a study by Ying *et al.* [5] and follow-up studies [10], [26], researchers found that neuroectodermal differentiation is not a default path, but rather that there is an obligate requirement for FGF4, typically produced by the cells themselves in an autocrine fashion, in the initiation of differentiation leading to neuroectodermal specification. This autocrine loop is sufficiently active at clonal density, as isolated mESCs are competent to form neural precursors [5], [12], [27]. Although no other autocrine\paracrine loops have been identified in neuroectodermal specification of mESCs, it is not known whether the FGF4 loop is the only such loop active in this process; in other words, whether FGF4 autocrine signaling is *sufficient* for neural specification of mESCs.

Making use of flow we have, for the first time, examined the question of sufficiency of FGF4 signaling in generating neuroectodermal precursors. We find that the primary role of FGF signaling is in acquiring neuroectodermal identity and that another autocrine\paracrine loop is required for growth during differentiation, leading to the conclusion that FGF4 is not sufficient for creating neuroectoderm. Our results demonstrate that perfusion can remove cell-secreted factors and affect diffusible signaling to the extent that significant effects on cell fate are observed. These findings establish microfluidic perfusion culture as a valuable method for investigating autocrine and paracrine signaling in biology.

## Results

### Device design

**Figure 1:**
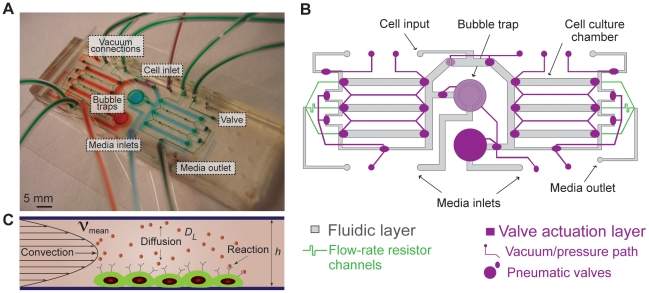
Microfluidic system. A. Image of the microfluidic perfusion device, showing the upper pneumatic control layer (green), the two sets of triplicate culture chambers (red and blue). B. Schematic of the perfusion device. Gray and purple outlines represent fluidic and control layer, respectively. C. Microfluidic perfusion systems use flow to fine-tune the relative significance of convection, diffusion, and reaction.

To modulate diffusible signaling, we developed a microfluidic perfusion platform that subjects cells to continuous medium flow while washing away cell-secreted factors (Figure 1A–B). The two-layer device incorporates multiple culture chambers and normally closed valves [28] that allow selective seeding of stem cells into culture chambers only and permit cell attachment in the absence of flow (Figure S1, S2). Active integrated bubble traps [29] prevent failure due to bubble introduction, permitting robust long-term culture of mammalian stem cells. We designed the microfluidic chambers to be 250 µm high and used a perfusion flow-rate of 33 µL/hr, which we have previously shown [30] is sufficient for robust growth of mESCs. The device culture chambers have a mirror symmetry design with the respect to the cell input to ensure balanced cell loading. Selective seeding of cells only into the culture chambers provides a well-defined culture system that minimizes conditioning of the media or nutrient depletion by cells that would otherwise be present in the fluid path upstream of culture chambers. Additionally, the cell loading path bypasses the integrated bubble traps, which are inline with the media inputs, to avoid cell settling in large areas due to the reduced fluid velocity present there (Figure S1). After loading, closing the valves to shut off fluid flow eases cell attachment for challenging cell types like ESCs. Finally, the multiple chambers are arranged to allow two conditions to be run side-by-side, enabling the use of controls to remove artifacts due to microscale perfusion culture. Together, these features are critical for the use of microfluidic platform in studying diffusible signaling, and contribute to the overall robustness of the perfusion system.

### Qualitative transport modeling

The ability to control the soluble cellular microenvironment is enabled by the properties of microscale fluid flow, specifically the ability to tune the relative importance of convective, diffusive, and reactive transport (Figure 1C). In order to use perfusion to alter diffusible signaling, one must operate in a regime where the rate of convection of ligand dominates over the rates of diffusion and reaction (*i.e*, ligand binding), which can be estimated by the ratio of the dimensionless Péclet number, *Pe*, and Damköhler number, *Da* (note that we actually refer to the Damköhler group II [31], though we will use the more generic term Damköhler number since it is the only one used in this manuscript). We explored whether a typical cytokine/growth factor such as FGF-4 could be swept away in microfluidic perfusion. FGF-4 has a MW of ∼20 kDa and an estimated diffusivity *D_L_*∼10^−6^–10^−7^ cm^2^/s, which we obtained from standard scaling arguments for the diffusivity of macromolecules of different molecular weights [32]. To choose a conservative value that would, if anything, underestimate the importance of convection in relation to diffusion, we use *D_L_* at the higher end of the range (∼10^−6^ cm^2^/s).

We first examined the relative significance of convective and diffusive transport, as parameterized by the non-dimensional Péclet number, *Pe* = ν_mean_ *h*/*D_L_*, where *Pe*≫1 denotes convection-dominated transport (Figure 1C). Here, ν_mean_ is the mean fluid velocity in the chamber (∼0.03 mm/s), and *h* denotes the characteristic length in the system, which we take as the chamber height (250 µm). For these values, we find that *Pe*≈75, indicative of convection-dominated transport. This velocity and chamber height in turn imply a cell surface shear stress of ∼3×10^−3^ dyn/cm^2^, accounting for flow perturbations due to the presence of attached cells [33]. This shear is at least 100× lower than the minimum values reported to adversely affect ESC developmental potential or used in differentiation of mESCs in previous work [34], [35], [36].

To include ligand binding in our estimate, we employ the Damköhler number, 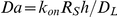, which compares the relative significance of ligand binding (*i.e.*, reaction) and diffusion of the ligand, where *k_on_* is the binding constant and *R_s_* is the receptor density. The number of FGF receptors per cell varies for different cell types from 700 receptors per cell in mouse myoblast MM14, to 20000 and 30000 receptors per cell for mouse 3T3 fibroblasts and mouse C3H10T1/2, respectively [37]; we chose as a reasonable estimate 10000 receptors/cell and a radius of the attached cells ≈10 µm. Values of ligand-receptor binding constant *k*_on_ vary greatly among ligand-receptor pairs, but prior measurements of binding of FGF ligands with their receptors have obtained values of *k*_on_ of 4.2×10^5^ M^−1^ s^−1^ for FGF2 binding to basement membrane [38], 1.4×10^5^ M^−1^ s^−1^ for FGF10 binding to FGFR2 [39], and 2.0×10^5^ M^−1^ s^−1^ for FGF1 binding to FGFR3c [40]. We chose a conservative estimate for *k*_on_ of 10^6^ M^−1^ s^−1^ (>2× higher than literature values), which would if anything overestimate the importance of ligand binding (and in turn decrease the importance of convection). These parameter choices result in *Da*∼0.132, implying that diffusion dominates over reaction. The ratio of these two dimensionless numbers, *Pe* and *Da*, will estimate the relative significance of convective and reactive transport, and we find that *Pe/Da* will be ≫1 (∼500), implying that microfluidic perfusion has the ability to suppress to a large extent diffusible cell signaling. We emphasize that these nondimensional parameter estimates do not prove that convection will remove cell-secreted factors from the system, but merely provide motivation and guidelines for system design.

### Effects of perfusion on neuroectodermal specification

**Figure 2:**
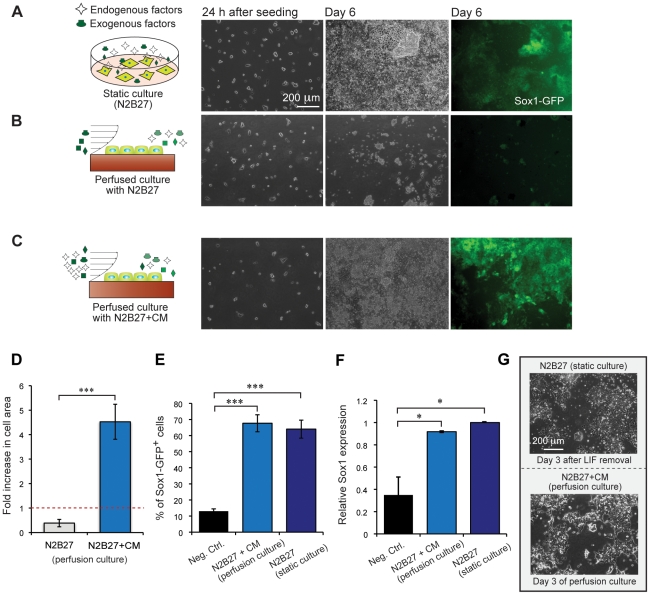
Monoculture neuroectodermal differentiation and comparison of differentiation in static and perfusion systems. (A–C) Schematic of culture conditions and images of 46C mESCs in different culture conditions taken 24 hours after seeding (left), and 6 days after attachment (middle and right) for (A) static differentiating cultures in N2B27 medium, (B) on-chip perfused culture in N2B27 medium, and (C) on-chip perfused culture in N2B27 medium with conditioned medium (CM). D. Fold increase in cell area after 5 days of perfusion culture for two different conditions, N2B27 and N2B27+CM. Data are average ± s.d. of 3 independent experiments. E. Analysis of Sox1 protein level - frequency of Sox1-GFP^+^ cells after 7 days of differentiation for N2B27+CM (perfusion culture) and N2B27 condition (static culture), assessed via flow cytometry. Data are average ± s.d. of 3 independent experiments. F. Analysis of gene expression for N2B27+CM condition in perfusion culture - relative Sox1 gene expression for N2B27+CM (perfusion culture) on Day 7 of differentiation normalized to GAPDH and gene expression level of Sox1 for N2B27 condition (static culture). Data are average ± s.d. of 2 independent experiments. 46C mESCs in self-renewal condition (N2B27+LIF+BMP4) were used as a negative control for both flow cytometry and qRT-PCR analysis, (* indicates P<0.05; *** indicates P<0.001). G. Representative phase images of mESCs colonies undergoing differentiation for three days in static and perfused cultures.

To investigate the role of cell-secreted factors in neuroectodermal differentiation of mESCs, we differentiated cells to neuroectoderm in serum-free conditions, using a Sox1-GFP^+^ (46C) mESC cell line to report on differentiation status, where Sox1 is the earliest known marker of neuroectoderm in the mouse embryo [41]. mESC neuroectodermal differentiation is well-suited for study of diffusible signaling using perfusion because (1) chemically-defined (serum-free) self-renewal and neuroectodermal differentiation conditions have been identified [42], avoiding confounding effects of the unknown factors introduced by serum; (2) the cells can be cultured and differentiated in adherent monoculture consisting of ∼2–3 layers of cells, giving the fluid flow easy access to the cell surface for altering transport; (3) fluorescent reporter cell lines are readily available, in contrast to human ESC cultures; (4) the existence of the FGF4 autocrine loop required for neuroectodermal differentiation provides a test case for the microsystem; and (5) biological insights derived from mESCs can often be applied to hESCs. Cells cultured in static conditions in dishes had by day 6 undergone differentiation into Sox1-GFP^+^ neural precursors, as expected [5] (Figure 2A). In contrast, cells cultured at equivalent areal densities in microfluidic perfusion in the same medium (N2B27) had very few cells by day 6 (Figure 2B).

The lack of growth in the perfused N2B27 condition could be due to alteration of the diffusible environment, shear-induced growth alteration, or some artifact due to culture in the microsystem (such as nutrient depletion). To examine these possibilities, we supplemented the N2B27 medium with cell-secreted factors obtained from the differentiating cultures (N2B27+conditioned medium (CM)). CM was conditioned in static dishes, and collected on day 3 after LIF withdrawal, which temporally correlates with the initial emergence of neural precursors, and dialyzed against fresh media to compensate for changes in the small-molecule fraction of the media, *i.e.*, due to nutrient depletion. The resulting CM would have the same nutrient concentrations as fresh media and similar volume- and time-average secreted molecule concentrations as the static control. Use of this media rescued the ability of the cells to grow and differentiate (Figure 2C–D). Additionally, cells perfused with CM attained similar morphology, Sox1-GFP levels, and Sox1 mRNA expression as static controls differentiated in N2B27 (Figure 2E–G), and did not preferentially differentiate into non-ectodermal lineages (Figure S3A) or self-renew (Figure S3B). Because cells perfused in both N2B27 and N2B27+CM experienced the same shear and nutrient delivery and are grown in the same microsystem, the difference in outcomes strongly precludes a role for shear stress, nutrient delivery, or microsystem artifact in our results, and strongly suggests that the microfluidic system is indeed sweeping away cell-secreted factors, which are then reintroduced in the CM. Further, these results argue for the presence of autocrine and/or paracrine factor(s) that contribute to growth of differentiating mESC cultures, *i.e.*, mESC differentiation (in N2B27) is not exclusively attributable to exogenously added factors, but rather requires cell-secreted factors. Notably, these diffusible factor(s) do not have anti-neurogenic activity, since in our cultures they are added continuously over 5–7 days of differentiation and not only promote growth of cells (Figure 2C), but also result in a frequency of Sox1 expression comparable to that of mESCs differentiated in static conditions in N2B27 (Figure 2E). Thus, our results provide strong evidence that microfluidic perfusion can alter diffusible signaling to affect neuroectodermal differentiation of mESCs.

### Sufficiency of FGF4 signaling during neuroectodermal differentiation

In examining which autocrine\paracrine loops might be being perturbed in perfusion, we first focused on FGF4, a cell-secreted factor known to be required for neuroectodermal specification. Confirming the role of FGF4 in neuroectodermal specification, we observed that blocking FGF signaling (by a small molecule FGF receptor inhibitor (FGFRi) - PD173074) in both static culture conditions N2B27 and N2B27+CM reduced differentiation but not growth (Figure S4), as others have observed [5]. As these static experiments block only FGF signaling while leaving all other diffusible signaling active, they cannot elucidate whether additional (non-FGF) autocrine\paracrine loops are also involved in neural specification.

**Figure 3:**
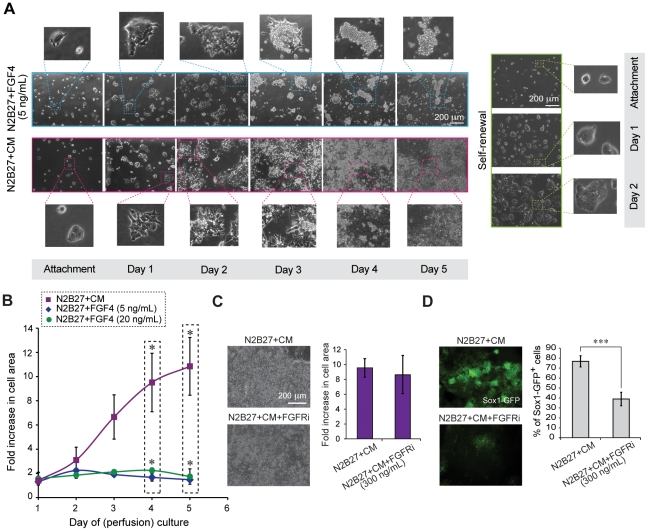
FGF signaling in perfusion culture. A. Phase images and close-ups of representative mESC colonies growing under differentiation and self-renewal conditions. Representative images of time-course changes in mESC morphology under two differentiation conditions in perfusion culture (N2B27+FGF4 and N2B27+CM) (left) or under self-renewal conditions in static culture (serum+LIF) (right). B. FGF4 supplementation in perfused mESC differentiation. Growth curves for cells differentiated in N2B27+CM vs. N2B27+FGF4 (5, 20 ng/mL) in perfusion for 5 days. Data shown are average ± s.d. of 2 independent experiments for each FGF4 concentration (* Indicates P<0.05). (C–D) Inhibition of FGF signaling in perfused mESC differentiation cultures. C. Transmission images of cells cultured in perfusion for 6 days in N2B27+CM and N2B27+CM+FGFRi (300 ng/mL) (left). Growth analysis for cells cultured in the presence of FGFRi at 300 ng/mL. Fold increase in cell area after 5 days of perfusion culture for two different conditions, N2B27+CM and N2B27+CM+FGFRi (right). D. Fluorescence images of cells cultured in perfusion for 6 days in N2B27+CM and N2B27+CM+FGFRi (300 ng/mL) (left). Sox1-GFP^+^ cell frequency assessed by flow cytometry for cells differentiated in N2B27+CM and N2B27+CM+FGFRi condition on Day 6 of perfusion culture (right). Data are average ± s.d. of 3 independent experiments, (*** Indicates P<0.001).

To investigate whether additional loops are involved, we supplemented N2B27 with FGF4 in perfused cultures, using N2B27+CM as a positive control. Cells initially attached at similar amounts in both conditions (for instance, for one representative experiment the attachment area was ∼7.5% (±0.87%) of the total chamber area for N2B27+FGF4 cultures and ∼7.3% (±2.3%) for N2B27+CM cultures 24 hours after seeding). On days 1 and 2, cells appeared to grow similarly in both conditions (Figure 3A), and exhibited similar morphologies that were distinct from the morphology of cells grown in self-renewal (Figure 3A). By day 3, however, cell growth in the presence of FGF4 started to deviate from that in the CM-supplemented condition (Figure 3A–B), and by day 5 cell growth in N2B27+CM was more than 6-fold greater than in N2B27+FGF4 (Figure 3B); FGF4 supplementation thus failed to rescue growth of long-term cultures on chip. Increasing the concentration of FGF4 4-fold did not restore growth under perfusion (Figure 3B). These data strongly suggest that FGF4 is not sufficient for promoting growth of differentiating mESCs to neuroectoderm, and suggest an important role for other cell-secreted factors during the time course of neuroectodermal specification.

### Time course of action of diffusible factors

To determine whether these other soluble factors in CM act on both growth and differentiation or only growth or differentiation, we blocked FGFR signaling under perfusion. We added the FGFRi PD173074 to cells cultured in N2B27+CM in perfusion at concentrations known to inhibit differentiation in static cultures (Figure S4A–B), and compared growth and Sox1 expression to control perfused cultures in N2B27+CM without the inhibitor. After 6 days of perfusion, cells in both conditions reached confluency (Figure 3C), demonstrating that inhibiting FGF signaling did not significantly affect growth of mESCs. However, there was a significant decrease in the frequency of Sox1-GFP positive cells when we added the FGFR inhibitor (Figure 3D, Figure S5). These results confirm that the role of FGF4 in the process of neuroectodermal specification of mESCs is primarily tied to the regulation of differentiation and acquiring neuroectodermal identity, and show that the other ligands in CM are responsible for survival or growth.

**Figure 4:**
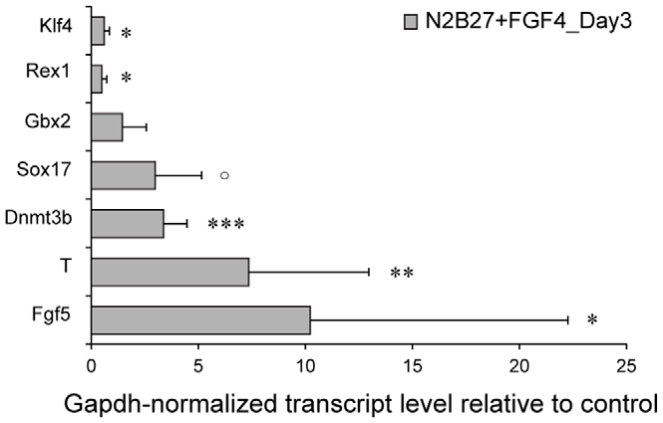
Gene expression profiling of committed mESCs in perfusion. Relative expression of genes expressed in mESCs (Klf4, Rex1) and epiblast (Sox17, Dnmt3b, T, Fgf5) for cells in perfusion culture (N2B27+FGF4 at 5 ng/mL), normalized to GAPDH and to self-renewal static culture (N2B27+LIF+BMP4). Data are shown as average ± s.d. from 4 independent experiments, (* Indicates statistical significance, ° P = 0.06, * P<0.05, ** P<0.01, *** P<0.001 for logarithmic distribution). The large variability in FGF5 gene expression is typical for this gene [26].

**Figure 5:**
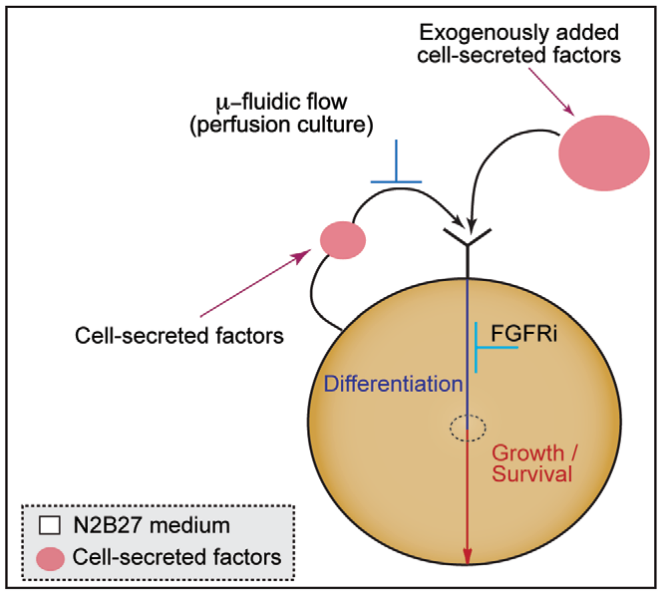
Graphical model of signaling in mESC neuroectodermal differentiation. Removing cell-secreted factors suppresses growth and differentiation, which can be restored by supplementing N2B27 medium with cell-secreted factors. Supplementation with (cell-secreted) FGF4 does not rescue growth of perfused mESC cultures, while inhibition of FGF signaling downregulates differentiation without compromising mESC growth.

Recent evidence suggests that ESCs attain a neural fate in two stages, first progressing thru an epiblast-like “primed” stage and then to Sox1^+^ neural precursors [43], reminiscent of the *in vivo* transition from ICM to epiblast to neuroectoderm. Since progression through the epiblast-like stage occurs ∼2–3 days into differentiation, which is approximately when we observe differences between CM-supplemented and unsupplemented medium (Figure 3A–B), we investigated the possibility that the requirement for these non-FGF cell-secreted factors occurs for cells in an epiblast-like state. Indeed, cells obtained from cultures 3 days in perfusion had downregulation in self-renewal markers Klf4 and Rex1 and upregulation of epiblast markers Fgf5, T, Sox17, and Dnmt3b [44], [45], [46], [47], [48] (Figure 4), which is supportive of this two-stage model. Together, our findings suggest a scenario where diffusible factors act downstream of FGF4-induced lineage commitment to regulate the growth of committed cells (Figure 5).

## Discussion

We have utilized microfluidic perfusion to investigate the role of autocrine and paracrine signaling in cellular decision processes, and specifically in the differentiation of mESCs into neuroectodermal precursors. Underlying this approach is the concept that microfluidic perfusion allows operation in a convection-dominated transport regime. Although it is likely that not all cell-secreted factors are fully swept away in flow-based systems, even in convection-dominated transport (because the *Pe* number is not infinite and because binding of cell-secreted factors to extracellular matrix can compete with convection), our results show that perfusion can significantly alter diffusible signaling. The fact that cells in perfusion culture did not survive under culture conditions with reduced soluble factors (N2B27), whereas supplementing N2B27 with cell-secreted factors (N2B27+CM) both fully recovered growth as well as allowed differentiation into neuroectoderm, point to a role for diffusible factor(s) in sustaining mESC growth in neuroectodermal differentiation. We then examined whether previously identified autocrine FGF4 was the causative agent being removed in perfusion by studying *two extremes*: supplementing minimal medium N2B27 with FGF4, and inhibiting FGF signaling in fully supplemented medium (N2B27+CM). These results strongly suggest that FGF4 does not act alone, that other autocrine\paracrine factors are involved in cell growth during differentiation. To our knowledge, our results identifying the existence of another autocrine\paracrine loop in neural specification are the first instance that microfluidic perfusion has been used to demonstrate a biologically relevant outcome in a cell system operating under conditions of reduced autocrine\paracrine signaling.

Interpreting results obtained using microfluidic perfusion requires care because of possible confounding factors. First, because the surface-area-to-volume ratio of the microchambers (4/mm) differs significantly from conventional cultures (∼0.75/mm for a 10-cm dish), plating cells at the same areal density in both systems will result in a higher cell volumetric density in the microsystem [49], potentially leading to nutritional artifacts. These can be avoided by perfusing fast enough to adequately feed the cells, but typifies the challenge in interpreting phenotypic differences between conventional static and perfused microfluidic (or static microfluidic) cultures, and is the reason why the different phenotypes observed in Figure 2A versus Figure 2B cannot be easily ascribed to a cause.

The other confounding issue is that of shear stress. The convection needed to alter diffusible signaling brings along fluid shear, which can affect cell phenotype [34], [35], [36]. Operating at shear stresses ≪1 dyn/cm^2^ (here we use 0.003 dyn/cm^2^), significantly below those known to affect cell phenotype, helps reduce the likelihood of shear stress effects. Perhaps the best way to disambiguate the effects of shear from soluble signaling, though, is through the use of perfusion with CM (*e.g.*, Figure 2C). By comparing outcomes between experiments with the same flow-rate but different soluble environments (Figure 2B vs. Figure 2C), the difference in observed phenotypes can be attributed to the absence\presence of the soluble signals. The primary caveats in interpreting CM experiments are that (1) failure to restore phenotype with a CM experiment does not prove that diffusible signaling is not important, because short-lived soluble factors may not survive in CM and because the CM contains the average ligand concentrations, which may be different (and generally lower) that those present locally in the static culture system, and (2) it is formally possible that the phenotype observed with perfused CM is due to the combination of shear and restored soluble factors. In our experiments, since we are able to restore phenotype with CM (Figure 2C), the first issue is not of concern. Additionally, it is unlikely that our restored differentiation (Figure 2C) is due to the combined action of shear and diffusible signaling because the phenotype is qualitatively and quantitatively similar to that observed in the absence of shear (Figure 2E–G); since soluble factors with shear (Figure 2C) and soluble factors without shear (Figure 2A) have similar phenotypes, the likeliest explanation is that shear has negligible effect in our system.

Instead of CM experiments as performed here, one could alternatively use a recirculating loop to let the cells condition the media directly, however (1) recirculating loops are difficult to construct at the microscale because the loop volume needs to be similar to the culture volume (∼12 µl), and (2) recirculating loops do not allow for media exchange and as a result nutrients may become limiting. Thus, observed phenotypes in recirculating loop experiments could be due to diffusible signaling and/or metabolic processes. Similarly, experiments that vary flow-rate as a means to vary diffusible signaling also vary nutrients and shear, making it difficult to disentangle the contributions of diffusible signaling from other effects. Thus, CM experiments, when they are able to restore phenotype, provide the cleanest experimental interpretation.

Our findings suggesting that FGF4 is not the sole cell-secreted factor responsible for neuroectodermal specification, but rather that other factors are involved in promoting growth during differentiation, are consistent with the previous reports regarding the distinct roles of FGF4 in promoting proliferation and differentiation of embryonic stem cells and their differentiated progenies. Kunath *et al.* used a variety of mESC knockout lines and small molecule inhibitors to identify FGF4 as a pivotal activator of Erk1/2 signaling in undifferentiated mESCs, and showed that inhibition of Erk1/2 or FGFR signaling did not disturb the expansion of undifferentiated ES cells but rather impaired their ability to commit to neural and mesodermal lineages [10]. Wilder *et al.* derived FGF4^−/−^ mESCs and also reported no requirement for FGF4 for growth of mESCs, but noted reduced numbers of differentiated cells when mESCs were cultured in the absence of FGF [50]. They ascribed this result to a growth-supportive paracrine effect of FGF4 on differentiated cells, but in light of more recent results [10], [26], this could also be due to differential growth of differentiating mESCs versus mESCs with a block in neural commitment. Thus, current understanding is that FGF4 has no effect on mESC growth or self-renewal, but is important for initiation of differentiation into neuroectoderm [51]. However, these studies do not examine sufficiency of diffusible FGF4 signaling, as they do not remove *all* autocrine\paracrine signaling, leaving open the possibility that FGF4 acts in concert with other factors in promoting this process.

The presence of non-FGF autocrine\paracrine loop(s) has thus far been obscured in traditional culture settings, even though robust neural differentiation protocols have existed for >15 years [24] and are employed routinely in the literature. Because neural differentiation is successful at clonal density in static culture [5], [12], [27], any autocrine\paracrine loops involved are sufficiently active at clonal density (including the FGF4 autocrine loop in mESCs, which is almost fully saturated at clonal density [5]). Thus, the effects of these loops would not be observed by varying plating density, because if a single-cell produces enough ligand to activate the loop, then increasing cell density would only increase the ligand concentration and would not reveal their existence. Similarly, the use of CM in static culture mimics increased density and will be similarly uninformative. Instead, one way to approach this question in static culture would be to develop a loss-of-function screen (*e.g.*, RNAi) to identify factors required for neuroectodermal specification, and follow-up studies of hits could pinpoint factors acting in an autocrine\paracrine fashion.

More generally, our results demonstrate the utility of perfusion as a biophysical tool to interrogate diffusible signaling in a more defined culture setting, complementing existing approaches that modulate known autocrine\paracrine loops, using ligand addition (*e.g.*, FGF4 supplementation) and receptor inhibition (*e.g.*, FGFRi). Because perfusion nonspecifically disrupts diffusible signaling, it allows for more stringent identification of sufficiency of extrinsic factors for cell processes than is possible in a static culture. Together, these methods constitute a useful screening strategy to identify candidate cell-secreted molecules by sequential subtraction/inhibition of signaling molecules in fully supplemented medium and addition of the same signaling molecules to putative minimal/sufficient medium. Beyond stem cell biology, the methodology we present offers a novel tool for studying other autocrine\paracrine systems, and could serve as a screening strategy for identifying the cohort of extrinsic molecules involved in diverse cellular processes.

## Materials and Methods

### Microfluidic perfusion device

The microfluidic perfusion device is a two-layer PDMS device that consists of a fluidic layer (the bottom layer sealed to the cell attachment substrate) controlled by normally closed valves actuated via a pneumatic layer (the top layer). The pneumatic layer consists of 100 µm-high displacement chambers connected with 100 µm-high channels that can selectively be actuated with vacuum (valves opened), or pressure (valves closed). The fluidic layer consists of two bubble traps, two sets of three 250 µm-high×13000 µm-long×1250 µm-wide cell culture chambers, and corresponding flow-rate setting resistor channels at the outlet of the chambers (100 µm width×100 µm height) of the same length to achieve equal flow-rate distribution across the different chambers. Fluid paths are altered by selectively actuating different valve combinations throughout different stages of the experiment (Figure 1B, Figure S1).

3-D AutoCad drawings were used to generate 3-D plastic molds of the pneumatic and fluidic layer (Fineline, NC). Before device fabrication, molds were silanized for 24 h in a vacuum chamber with tridecafluoro-1,2,2-tetrahydrooctyl)-1-tricholorosilane (T2492-KG, UnitedChemical Technologies, PA). Polydimethylsiloxane (PDMS, Sylgard 184, Dow Corning, Midland, MI) was poured onto both featured molds (10∶1 ratio of prepolymer base to curing agent). After pouring PDMS, the fluidic layer was covered with a transparency film and then the entire assembly was placed between two aluminum plates and clamped. This, along with designed 250 µm high cell culture chambers, and the support pillars (500 µm height) ensured the overall thickness of the fluidic layer to be 500 µm, and those of cell culture chambers and the actuating membrane to be half the size (250 µm). Both PDMS layers were left overnight to cure at 65°C, and removed from the molds afterwards. The featured side of the pneumatic layer (with the displacement chambers) and blank side of the fluidic layer were plasma cleaned (PDC-001, Harrick Plasma, Ithaca, NY), manually aligned, and bonded together. The assembled device was left overnight at 65°C to thermally strengthen the bond. Pneumatic connections were punctured through the pneumatic layer only before bonding, using thin-walled tubing (0.07″ od×0.0653″ id, Small Parts Inc.). Fluidic connections were punctured through both layers, using the same tubing, after the bonding.

### Perfusion experiments: Device setup and perfusion culture

A sterile device was sealed to a sterile tissue culture polystyrene slide (260225, Ted Pella, Inc., Redding, CA), and clamped into a custom-designed microscope stage using three adjustable aluminum clamps (Figure S6A). A custom-made acrylic plate was added in-between the PDMS device and aluminum clamps to ensure equal pressure distribution and avoid collapse of the fluidic and pneumatic channels under clamping pressure. It also ensured a proper sealing of a device keeping the valves functional and avoiding any fluidic leakage. Sterile tubing (UpChurch Scientific, WA) was connected to the two media inputs, 4-way valve and tubing to the cell input, and 4 tubings connecting outlets to the waste tubes (Figure S6B).

A device was primed with 0.1% gelatin to insure bubble free device for cells loading and to have cell culture chambers coated with the gelatin to promote cell attachment. The following day, the device was firstly perfused with a culture medium to avoid perfusing on-chip cell culture with 0.1% gelatin and deprive cells from nutrients in the beginning of perfusion culture, placed on an automated inverted microscope fitted with a stage incubator (In Vivo Scientific, St. Louis, MO), preheated to 37°C. Cells were dissociated, counted and transferred to a 3 mL syringe (309585, BD plastic), and loaded manually applying gentle pressure to cells-filled syringe and while keeping valves of culture chambers open and those on a fluidic path of cells to the culture chambers (see Figure S1 for valve combinations used throughout different stages of the experiment). Culture chambers were closed, and a pressure of ∼1 psi was applied to ensure valve sealing while flushing the rest of a device with media from syringes connected to the two media inlets (this procedure restricted cell growth to the cell chambers only). After the loading procedure, cells were left for about 30 minutes to settle down in chambers, and imaged using Metamorph software; and the device was moved to the incubator (37°C, 7.5% CO2) overnight to allow for cell attachment. During the attachment period all the valves were left normally relaxed (closed) to prevent any fluid movement in the device that could potentially have an adverse effect on cell attachment. Perfusion was resumed ∼24 hours after seeding. Ten images (phase and/or fluorescence) were acquired per chamber (3 chambers per condition), and used in subsequent image analysis. The media syringes filled with corresponding fresh media, were mounted on a syringe pump (Harvard Apparatus Pump 11 Pico Plus) outside the incubator and set to constant flow-rate of 0.1 mL/hr (∼33 L µL/hr per chamber). Filters (PN 4612 25 mm syringe filters with 0.2 µm membrane) were combined inline with the media inlet tubing to maintain sterility while device being disconnected for imaging and served as large bubble traps as well. The device culture chambers were imaged daily during each experiment (up to 5–7 days of perfusion culture depending on the experiment), and cells were harvested from the chip on the last day for subsequent flow cytometry or qRT-PCR analysis.

### Cell culture

Sox1-GFP knock-in (46C) mESCs, developed by Austin Smith's group [5], were routinely propagated without feeders in leukemia inhibitory factor (10 ng/ml, ESG1107, Chemicon, Temecula, CA)-supplemented GMEM-based ES medium: GMEM (11710035, Invitrogen, Carlsbad, CA), 10% ES-qualified fetal bovine serum (SH30070.03, Hyclone), 100 µM-mercaptoethanol (M7522, Sigma, St. Louis, MO), and 50 U/mL penicillin, 50 µg/mL streptomycin (15140122, Invitrogen). We cultured cells directly on tissue-culture plastic (150679, Nunc) in a 37°C humidified environment with 7.5% CO2. For maintenance of 46C culture, we dissociated cells in TrypLE Express (12604013, Invitrogen) every other day, and fed in days between.

### Neuronal differentiation

For monoculture differentiation undifferentiated mESCs were dissociated, spun down, resuspended into LIF supplemented differentiation medium (N2B27), and replated onto 0.1% gelatin (ES-006-B Embryomax ES qualified gelatin, Millipore)-coated tissue culture plastic dishes. Cells were plated at a density of 0.5–1.0×10^4^ cells/cm^2^ (both static and on-chip culture unless noted otherwise) in N2B27+LIF (LIF was added the first day to promote attachment, as others have done [27]). Cells were allowed to attach for ∼24 hours, washed twice with PBS (Phosphate Buffered Saline, 14190 Invitrogen) to remove residual LIF and transferred to N2B27 alone, or containing growth factors (FGF4 – fibroblast growth factor, 235-F4 Recombinant Human FGF-4, R&D systems), and inhibitor (FGFR inhibitor, PD173074, Calbiochem). After LIF removal, medium was replaced every other day (N2B27, N2B27+FGF4) or every day (FGFR inhibitor PD173074). This protocol was applied to static cultures only. Perfusion cultures were perfused constantly in all the different culture conditions, with a single daily interruption during the image acquisition.

### Conditioned medium preparation

Cells were plated onto 0.1% gelatin-coated dishes (168381, Nunc) following the protocol for neuronal differentiation. Medium was collected from cells undergoing neuronal differentiation on day 3 after LIF removal. To account for possible nutrient depletion, collected medium was spun down for ∼45 minutes at ∼3200 g (manufacturer's directions) using Amicon Ultra centrifugal filter unit with a 3 kDa filter (UFC900324, Millipore) until reduced to ∼3% of its original volume, then supplemented with fresh N2B27 media to reach the original volume. We focused on the large-molecule fraction of the medium (>3 kDa), as that fraction is likely to contain most common signaling molecules (cytokines).

### Image acquisition and processing

All the images were acquired on an inverted microscope (Zeiss Axiovert 200 M, Thornwood, NY) using a 10× objective with an automated stage (Ludl MAC 5000, Hawthorne, NY). We used Metamorph imaging software (Molecular Devices, Downingtown, PA) to acquire the raw images, which were later processed using Matlab (Mathworks, Natick, MA). Fluorencence images were acquired with FITC filter set using an ImagerQE camera (LaVision). Exposure time was set up using GFP negative ES cells (D3 cell line, used as well as a negative control for flow cytometry), making sure to always use the same exposure when comparing different conditions from the same experiment. Cell growth was determined by quantifying the fold increase in cell spreading area at a given time point over the initial area of the attached cells ∼24 hours after seeding and before resuming perfusion culture. Phase images across the entire three cell culture chambers per condition of the microfluidic perfusion device were acquired and then used to perform image processing. Briefly, phase image analysis algorithm consists of the following steps: extract bright morphological features, adjust contrast, threshold, convert to binary image, and fill holes to calculate the cell area in each image. The average cell area is obtained by averaging over the entire field of the cell culture chamber. Percentage of cells expressing Sox1 was assessed similarly, for a particular time point phase and fluorescence images are analyzed to quantify the total cell area and area of cells expressing Sox1, respectively. Finally, the ratio of Sox1 area over the total cell area gave the fraction of cells expressing Sox1.

### Flow cytometry analysis

Cells in static cultures were dissociated using TrypLE Express (12605-010, Invitrogen, Carlsbad, CA) for 3 minutes, quenched with media, spun down and resuspended in serum-free medium supplemented with Propidium Iodide (PI) solution (P4864-10ML, Sigma-Aldrich) at a concentration of 20 µg/mL. Cells in on-chip cultures were dissociated using a manually driven syringe (previously used for cell loading) through cell culture chambers, filled with different buffers in the following order PBS, 0.25% Trypsin-EDTA (25200-056, Invitrogen, Carlsbad, CA), and the culture medium, and spun down after collecting cells in a falcon tube. Cells resuspended in the medium supplemented with a PI solution were analyzed using a FACS LSR II (Becton Dickinson, San Jose, CA).

### qRT-PCR analysis

The protocol for harvesting cells from the on-chip cell culture was same as for flow cytometry. Cells from both static and perfusion cultures were resuspended in 350 µl cell lysis buffer for subsequent processing, after trypsinizing and spinning down. Total RNA was isolated using RNeasy® Plus Microkit (74034, Qiagen, CA). cDNA was synthesized with DyNAmo™ cDNA Synthesis Kit (F-470, Finnzymes, Finland) according to manufacturer's instructions. Quantitative real-time PCR reactions were set-up using DyNAmo™ SYBR® Green qPCR Kits (F-400, Finnzymes, Finland) and performed on a MJ Opticon 2 real-time PCR machine (MJ Research, MA). Quantification of transcript amounts were based on a standard curve established with cDNA converted from Stratagene® qPCR mouse reference total RNA (750600, Agilent Technologies, IL). The transcript level of each gene was normalized to corresponding Gapdh level for a particular sample. The primers used are listed in Table S1.

### Cell counting

For both types of assays, static and on-chip, cells were counted on a Z2 Coulter Counter (Beckman Coulter, Fullerton, CA) to obtain cell volume concentration, which was later converted to equivalent areal density.

### Statistical analysis

Statistical analyses were performed using un-paired two-tailed Student's t-test assuming samples of equal variance.

## Supporting Information

Table S1 Quantitative real-time PCR primer sequences.
(DOCX)

Figure S1 Typical operational modes of the device used in perfusion experiments. Valves are used in various combinations throughout different stages of the experiment. Arrows indicate direction of flow. (1) To load cells, valves are actuated such that all culture chambers are connected to the cell input without going thru the bubble traps. (2) Afterward, the valve actuation pattern is altered to permit flushing of cells in regions of the device except for the chambers. (3) To permit cell attachment, valves at the chamber inlets and outlets are closed, preventing any fluid flow and thus permitting cell attachment. (4) Finally, during culture, the valves are actuated such that each set of three chambers is perfused with a different media that traverses the bubble traps.
(TIFF)

Figure S2 Merged phase and fluorescence images of Oct4-GFP mESCs (Oct4 GFP^+^ ABJ1 line) after two days of culture in a device.
(TIFF)

Figure S3 Relative gene expression in static (N2B27) and perfused (N2B27+CM) differentiating mESCs cultures. A. Comparison of gene expression for early differentiation markers Gata4 (endoderm) and Nkx2.5 (mesoderm) between static and on-chip cultures. B. Relative gene expression of three genes associated with self-renewal, in static cultures in N2B27 and N2B27+LIF+BMP4 (N2B27+LB), and perfused cultures in N2B27+CM. Data are shown as average ± s.d. from 2 independent experiments, (* Indicates statistical significance, * P<0.05, ** P<0.01, *** P<0.001). Gene expression is normalized to GAPDH and N2B27 (static culture) and N2B27+LIF+BMP4 (static culture), in (A) and (B), respectively.
(TIFF)

Figure S4 Flow cytometric measurement of Sox1-GFP neuronal precursors frequency upon addition of FGFR inhibitor to both N2B27 and N2B27+CM in static cultures at different concentrations. A. Relative frequency of Sox1-GFP^+^ cells upon addition of FGFR inhibitor to N2B27 at 100 and 300 ng/mL. B. Relative frequency of Sox1-GFP^+^ cells upon addition of FGFR inhibitor to N2B27+CM condition at 100 and 300 ng/mL. For both conditions N2B27+FGFRi and N2B27+CM+FGFRi Sox1-GFP expression is normalized to Sox1 expression of N2B27 and N2B27+CM condition respectively. Data are average ± s.d. of 3 independent experiments for (A), and 2 independent experiments for (B), (* Indicates statistical significance, * P<0.05, ** P<0.01). Non-GFP expressing D3 mESC line used as a control in flow cytometry to set the gate.
(TIFF)

Figure S5 Sox1 activation in different conditions in perfusion. A. Expression of Sox1 protein assessed via image analysis for cells differentiated in perfusion in N2B27+CM and N2B27+CM+FGFRi. Data are average ± s.d. of 3 independent experiments, (* Indicates statistical significance, *** P<0.001). B. Flow cytometry profiles of Sox1 activation in N2B27+CM and N2B27+CM+FGFRi.
(TIF)

Figure S6 A. Photograph of a device clamped into the microscope stage. B. Schematic of the perfusion setup.
(TIFF)
