# Supplementary figures and images for: Microfluidic Perfusion for Regulating Diffusible Signaling in Stem Cells
Source: PLoS One. 2011 Aug 4;6(8):e22892. doi: 10.1371/journal.pone.0022892 (PMC3150375; doi:10.1371/journal.pone.0022892)

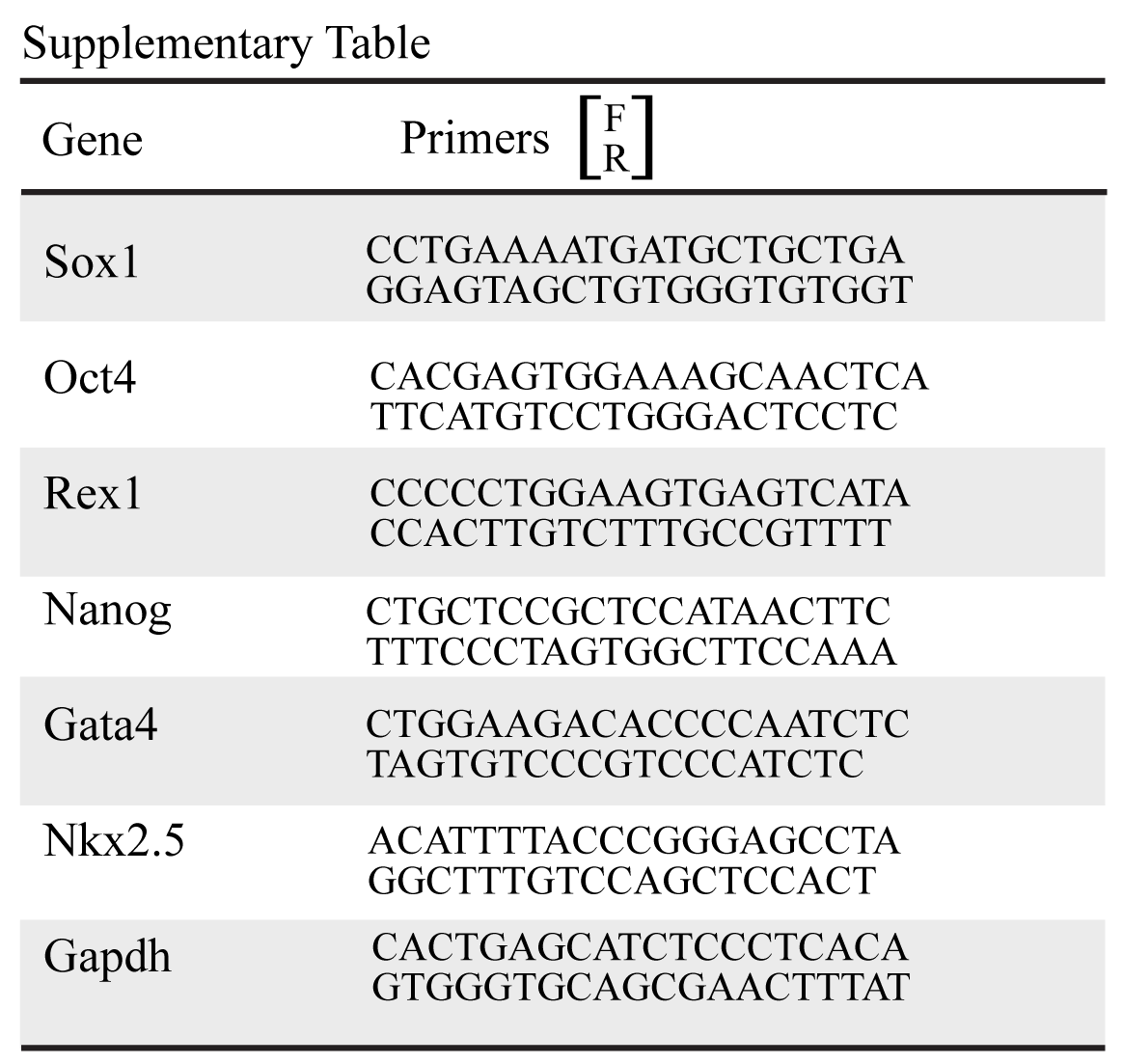


**Supplementary Table 1:** Quantitative real-time PCR primer sequences.

Supplement: Table S1 — Quantitative real-time PCR primer sequences. (DOCX) [file pone.0022892.s001.docx]

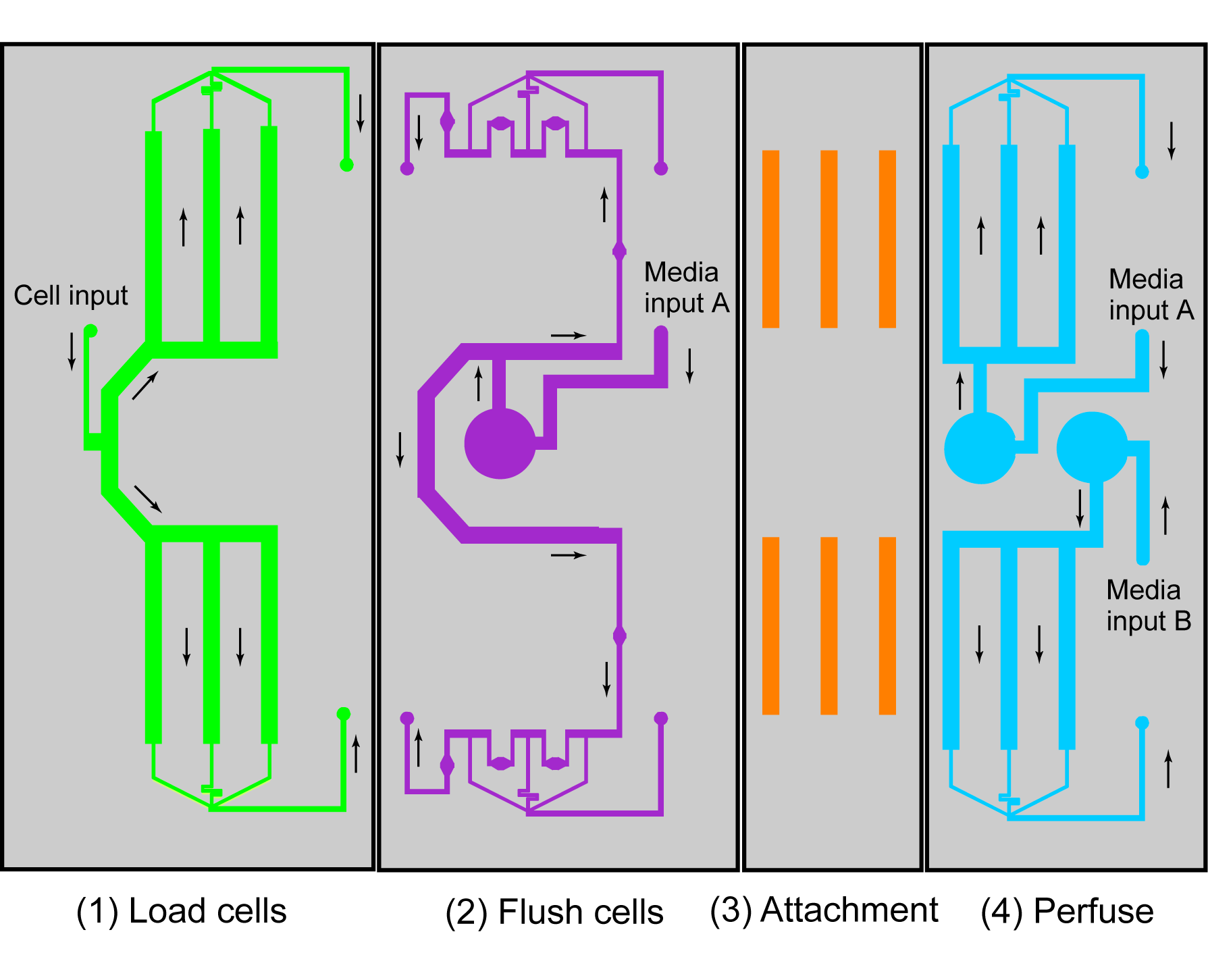

Supplement: Figure S1 — Typical operational modes of the device used in perfusion experiments. Valves are used in various combinations throughout different stages of the experiment. Arrows indicate direction of flow. (1) To load cells, valves are actuated such that all culture chambers are connected to the cell input without going thru the bubble traps. (2) Afterward, the valve actuation pattern is altered to permit flushing of cells in regions of the device except for the chambers. (3) To permit cell attachment, valves at the chamber inlets and outlets are closed, preventing any fluid flow and thus permitting cell attachment. (4) Finally, during culture, the valves are actuated such that each set of three chambers is perfused with a different media that traverses the bubble traps. (TIFF) [file pone.0022892.s002.tif]

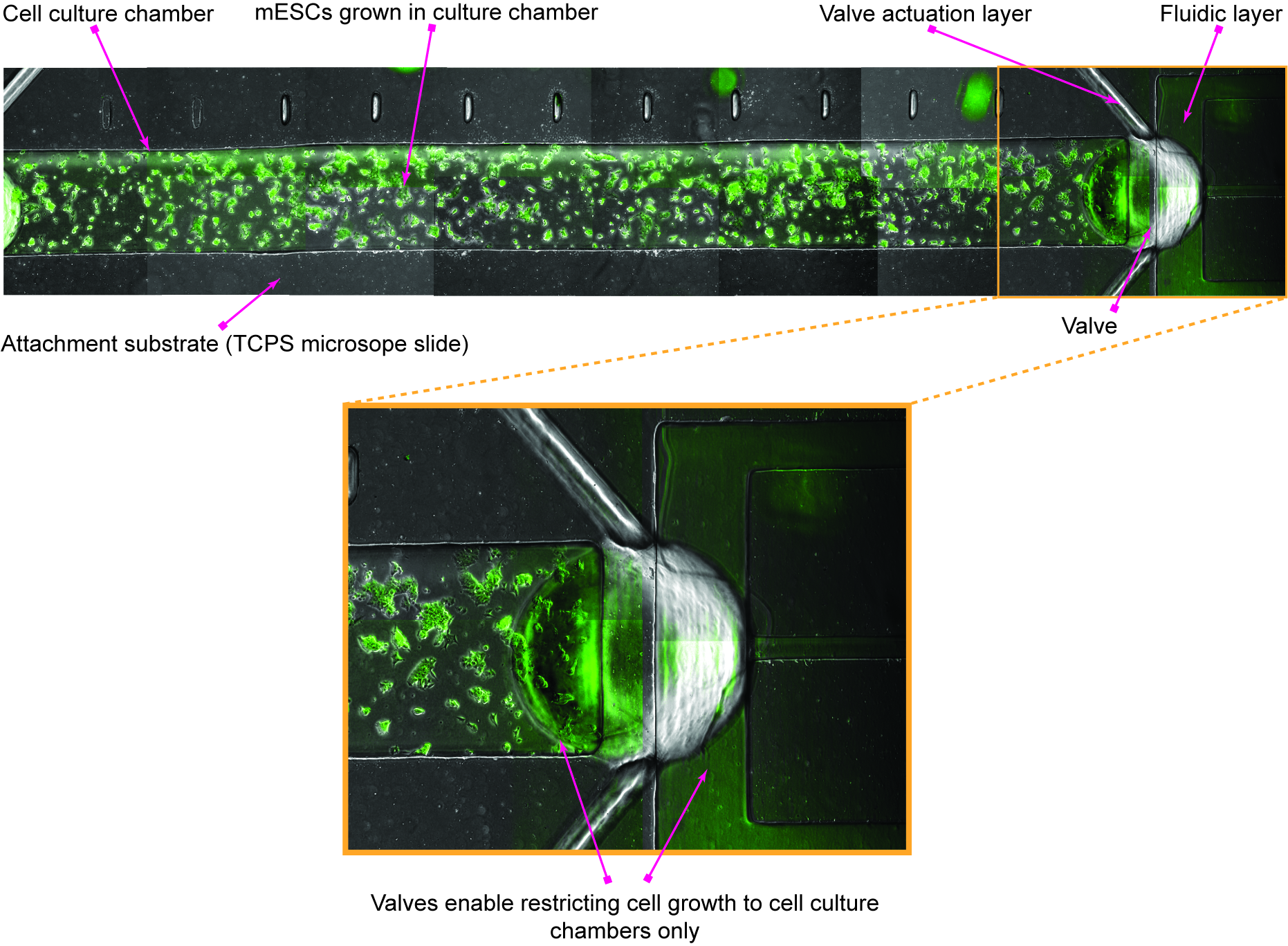

Supplement: Figure S2 — Merged phase and fluorescence images of Oct4-GFP mESCs (Oct4 GFP+ ABJ1 line) after two days of culture in a device. (TIFF) [file pone.0022892.s003.tif]

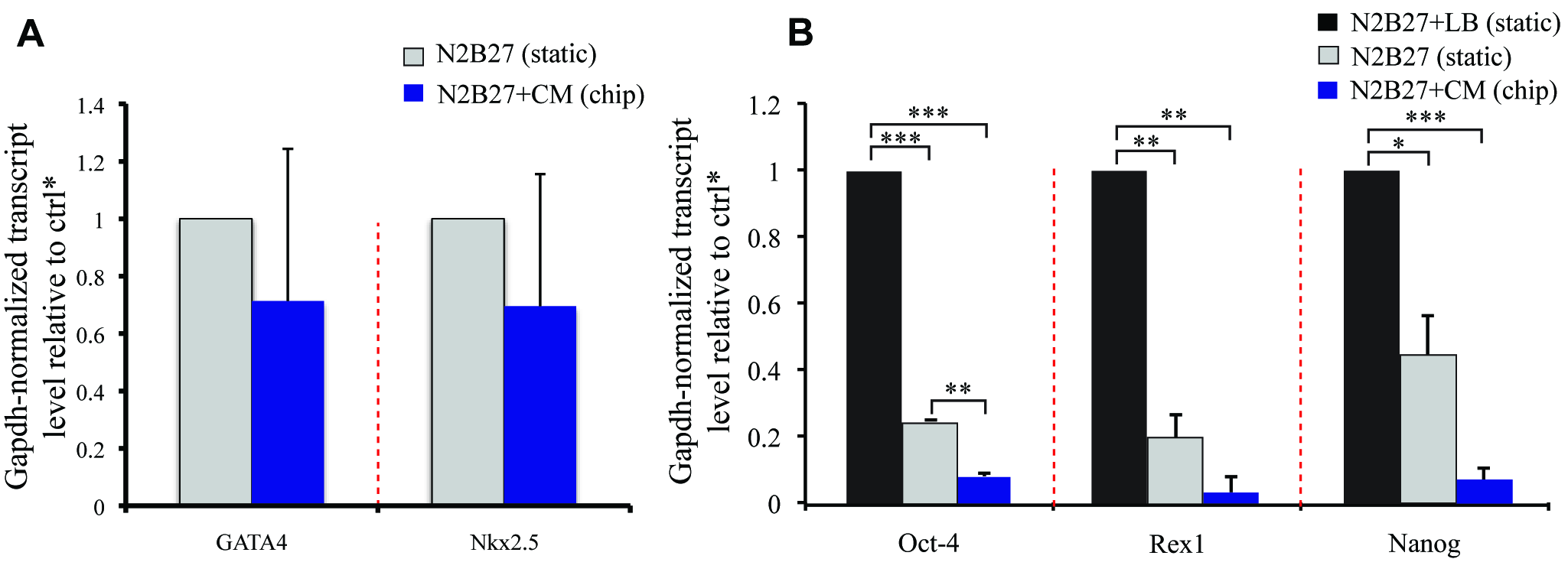

Supplement: Figure S3 — Relative gene expression in static (N2B27) and perfused (N2B27+CM) differentiating mESCs cultures. A. Comparison of gene expression for early differentiation markers Gata4 (endoderm) and Nkx2.5 (mesoderm) between static and on-chip cultures. B. Relative gene expression of three genes associated with self-renewal, in static cultures in N2B27 and N2B27+LIF+BMP4 (N2B27+LB), and perfused cultures in N2B27+CM. Data are shown as average ± s.d. from 2 independent experiments, (* Indicates statistical significance, * P<0.05, ** P<0.01, *** P<0.001). Gene expression is normalized to GAPDH and N2B27 (static culture) and N2B27+LIF+BMP4 (static culture), in (A) and (B), respectively. (TIFF) [file pone.0022892.s004.tif]

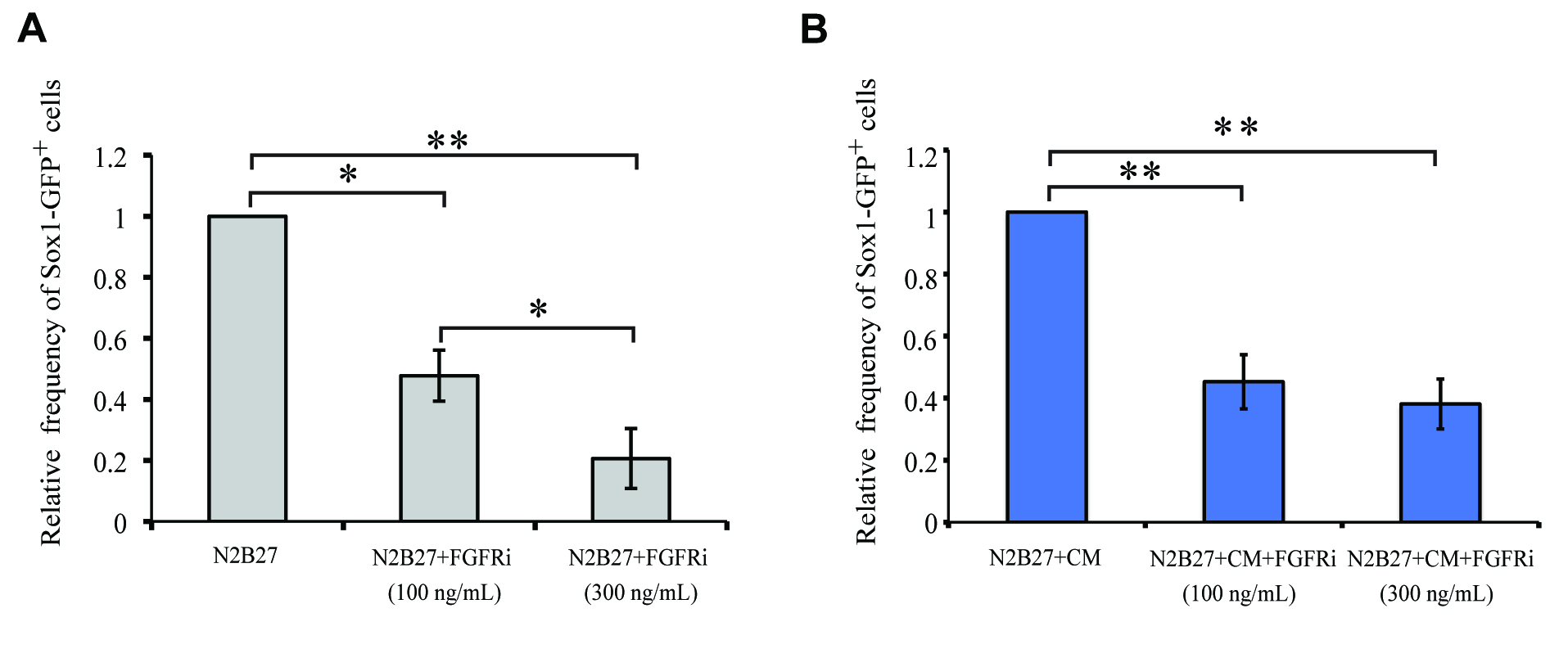

Supplement: Figure S4 — Flow cytometric measurement of Sox1-GFP neuronal precursors frequency upon addition of FGFR inhibitor to both N2B27 and N2B27+CM in static cultures at different concentrations. A. Relative frequency of Sox1-GFP+ cells upon addition of FGFR inhibitor to N2B27 at 100 and 300 ng/mL. B. Relative frequency of Sox1-GFP+ cells upon addition of FGFR inhibitor to N2B27+CM condition at 100 and 300 ng/mL. For both conditions N2B27+FGFRi and N2B27+CM+FGFRi Sox1-GFP expression is normalized to Sox1 expression of N2B27 and N2B27+CM condition respectively. Data are average ± s.d. of 3 independent experiments for (A), and 2 independent experiments for (B), (* Indicates statistical significance, * P<0.05, ** P<0.01). Non-GFP expressing D3 mESC line used as a control in flow cytometry to set the gate. (TIFF) [file pone.0022892.s005.tif]

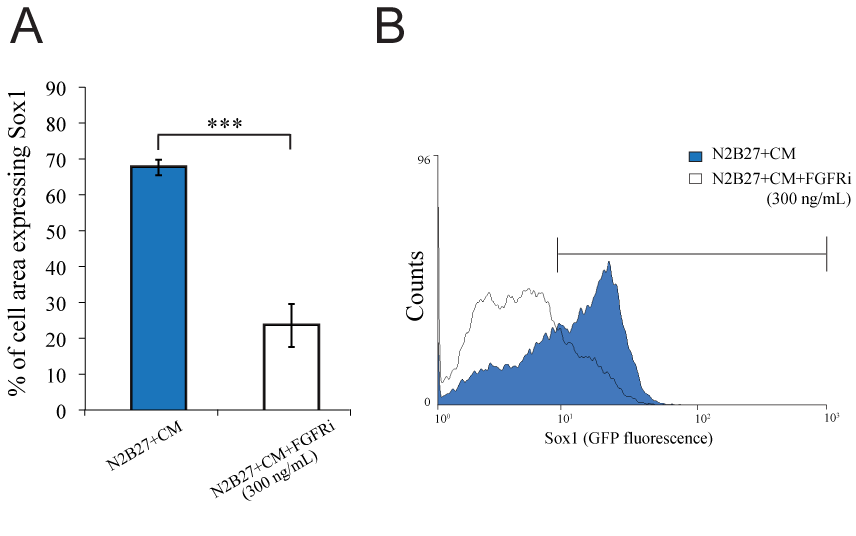

Supplement: Figure S5 — Sox1 activation in different conditions in perfusion. A. Expression of Sox1 protein assessed via image analysis for cells differentiated in perfusion in N2B27+CM and N2B27+CM+FGFRi. Data are average ± s.d. of 3 independent experiments, (* Indicates statistical significance, *** P<0.001). B. Flow cytometry profiles of Sox1 activation in N2B27+CM and N2B27+CM+FGFRi. (TIF) [file pone.0022892.s006.tif]

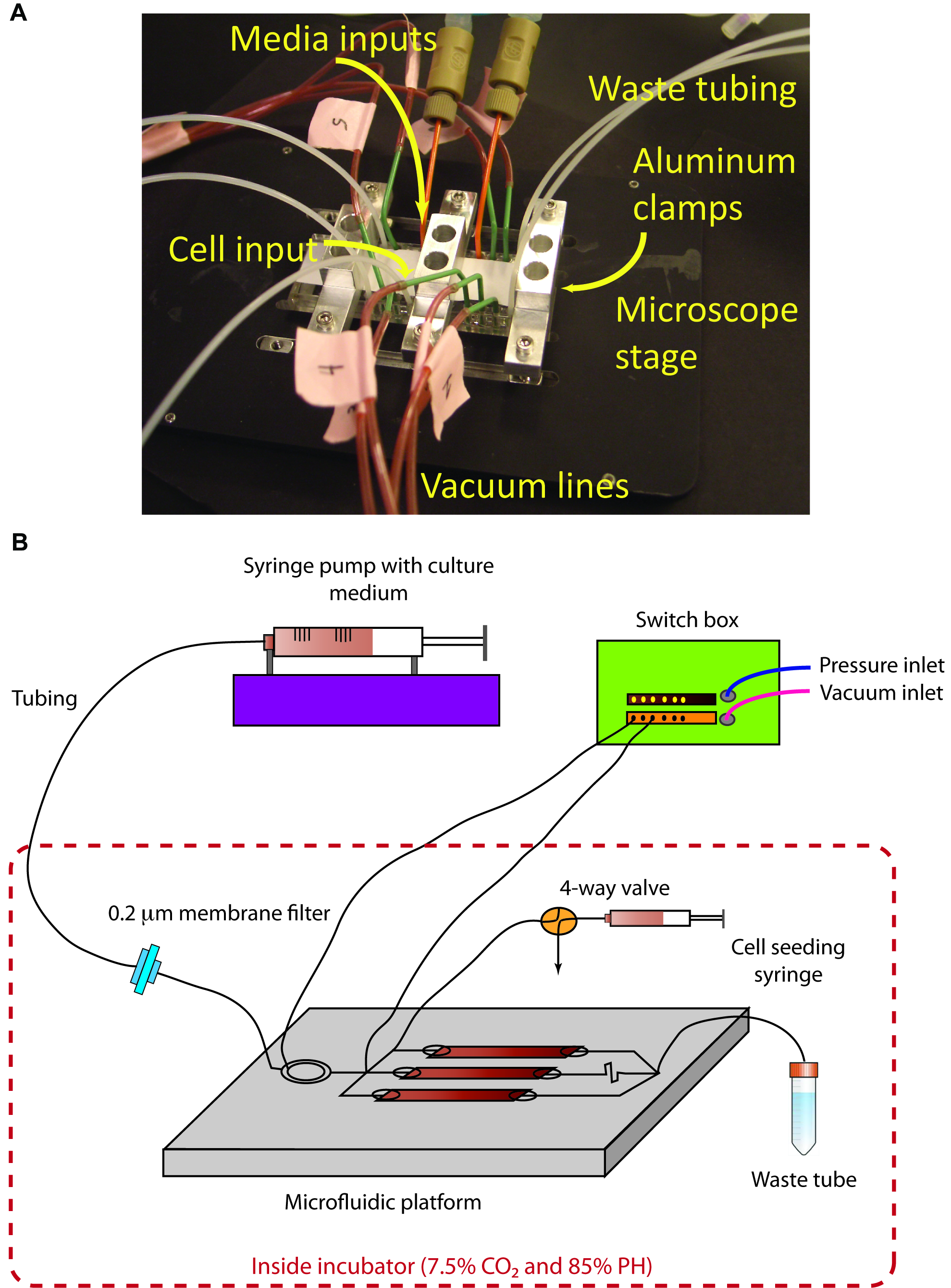

Supplement: Figure S6 — A. Photograph of a device clamped into the microscope stage. B. Schematic of the perfusion setup. (TIFF) [file pone.0022892.s007.tif]
